# Arterial structure and function in children with inflammatory bowel disease

**DOI:** 10.1002/jgh3.13100

**Published:** 2024-06-03

**Authors:** Asha Jois, Diana Zannino, Anthony G Catto‐Smith, Meg Kaegi, Jonathan P Mynard, Jeremy Rosenbaum, Mark Oliver, Winita Hardikar, George Alex, David Burgner

**Affiliations:** ^1^ Department of Gastroenterology and Clinical Nutrition The Royal Children's Hospital Melbourne Parkville Victoria Australia; ^2^ Clinical Epidemiology and Biostatistics Unit Murdoch Children's Research Institute Parkville Victoria Australia; ^3^ Department of Paediatrics University of Melbourne Parkville Victoria Australia; ^4^ School of Humanities, Arts and Social Sciences, University of New England Armidale New South Wales Australia; ^5^ Inflammatory Origins Group, Infection, Immunity and Global Health Theme Murdoch Children's Research Institute Parkville Victoria Australia; ^6^ Heart Research Group Murdoch Children's Research Institute Parkville Victoria Australia; ^7^ Biomedical Engineering, University of Melbourne Faculty of Engineering and Information Technology Parkville Victoria Australia; ^8^ Infectious Diseases Unit, Department of General Medicine The Royal Children's Hospital Melbourne Parkville Victoria Australia

**Keywords:** cardiovascular diseases, child, heart disease risk factors, inflammatory bowel diseases

## Abstract

**Background and Aim:**

People with inflammatory bowel disease (IBD) have an increased risk of cardiovascular disease, including in younger adulthood. This may arise in part from chronic, systemic low‐grade inflammation. The process of atherosclerosis may begin in childhood. We sought to determine whether pediatric IBD is associated with adverse changes in arterial structure and function as a marker of early increased cardiovascular risk.

**Methods:**

We performed a case–control study comparing children with IBD for a median disease duration of 2.49 (interquartile range 1.23, 4.38) years with healthy children. In a single visit, we collected baseline clinical and anthropometric data, and measured blood pressure, pulse wave velocity, carotid artery distensibility, and aortic and carotid intima‐media thickness. High‐sensitivity C‐reactive protein and fasting lipids were measured.

**Results:**

We enrolled 81 children with IBD (40 with Crohn's disease, 40 with ulcerative colitis, and 1 with unspecified IBD) and 82 control participants. After adjusting for age, sex, body mass index *z*‐score, blood pressure, and low‐density lipoprotein cholesterol, there was no difference in measures of arterial structure and function in children with IBD compared with controls, nor between those with Crohn's disease or ulcerative colitis.

**Conclusion:**

We did not show any differences in arterial structure and function in children with a history of IBD for less than 5 years compared with healthy controls. IBD diagnosed in childhood may provide a window of opportunity to actively reduce standard cardiovascular risk factors and improve future cardiovascular outcomes.

## Graphical abstract

As adults with inflammatory bowel disease (IBD) have an increased risk of cardiovascular disease, we sought to determine whether children with IBD might have early adverse changes in arterial structure function as a measure of increased cardiovascular risk. This case–control study found that after a mean disease duration of 2.49 years, children with IBD did not have a statistically significant increased risk of having abnormal measures of arterial structure and function.

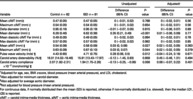

## Introduction

Inflammation is key to the pathogenesis of atherosclerosis,^1^ which develops asymptomatically across the life course and results in cardiovascular disease (CVD) in adulthood. CVD is the leading cause of morbidity and mortality worldwide.^2^ Patients with inflammatory bowel disease (IBD) have an increased risk of CVD.^3^, ^4^ This may be mediated by chronic, systemic, low‐grade inflammation, in addition to a hypercoagulable state and altered gut microbiota.^5^, ^6^

Noninvasive cardiovascular measures predict CVD in adults and are used to assess risk from childhood onwards.^6^, ^7^, ^8^, ^9^ These measures include blood pressure (BP), pulse wave velocity (PWV), carotid artery distensibility, and large arterial structure (aortic and carotid intima‐media thickness, IMT). Childhood is a critical period for detecting potentially adverse vascular phenotypes, allowing intervention before changes become irreversible.^10^

There is evidence to suggest preclinical atherosclerosis occurs in young adults with IBD, yet data from children are scarce and conflicting. This may reflect methodological issues, such as considering Crohn's disease (CD) and ulcerative colitis (UC) together.^11^, ^12^ In adults, IBD has been associated with increased PWV (indicative of increased arterial stiffness);^13^ however, two studies in children with IBD found no association.^14^, ^15^

To address these knowledge gaps, we aimed to determine whether pediatric IBD is associated with differences in arterial structure and function as a marker of increased CVD risk. We hypothesized that patients with pediatric IBD would have increased aortic and carotid IMT, increased PWV, and decreased carotid distensibility and compliance compared with healthy controls.

## Methods

### Study population

Participants were enrolled between October 2013 and May 2018. Children aged 5–18 years with a diagnosis of IBD for at least 6 months duration were eligible. Forty participants had a diagnosis of CD, and 40 had UC, with one participant with unspecified IBD (IBDU), as classified by their treating gastroenterologist. Participants were recruited through the gastroenterology outpatient service at the Royal Children's Hospital (RCH, Melbourne, Australia), a major tertiary pediatric hospital. Informed consent was obtained from participants' parent or guardian. Participants with IBD were excluded if their disease was limited to proctitis or perianal disease, or they were admitted to hospital within the preceding month. Eighty control participants, also between 5 and 18 years, were recruited through outpatient departments at the RCH or referred through case participants (non‐relatives) or staff members working at RCH or affiliated institutions. Informed consent was also provided for this group. Participants were excluded if they had an inflammatory comorbidity.

### Data collection

All participants were assessed at a single study‐dedicated visit. Demographic and anthropometric data (height, weight, waist, and hip circumference) were collected, using standard protocols described previously.^16^ Pubertal status was self‐reported using Tanner Staging. A blood sample was collected after a minimum 6‐h fast for measurement of high‐sensitivity C‐reactive protein (hsCRP) (Abbott Architect, IL, USA), glucose, triglycerides, total cholesterol, high‐density lipoprotein (HDL), and low‐density lipoprotein (LDL) cholesterol (Vitros 5600, Ortho‐Clinical diagnostics, NJ, USA).

### Cardiovascular measures

Participants were placed supine for three measurements of carotid femoral pulse wave velocity (cfPWV) with the SphygmoCor® XCEL device (AtCor Medical Pty Ltd., NSW, Australia) after a 5‐min rest, as per the manufacturer's protocol. Participants remained supine for three BP measurements using the same device. Ultrasound images of the carotid artery and abdominal aorta were taken using a portable ultrasound machine Vivid‐I (General Electronics Healthcare, Chicago, IL, USA), with simultaneous ECG gating as described previously.^17^, ^18^ Participants were supine with head turned to 45° for optimal carotid artery positioning. For carotid images, we recorded cine loops of at least five cardiac cycles for offline analysis, focusing on the intima‐media complex of the posterior wall of the right common carotid artery. Aortic images focused on the posterior wall, just proximal to the femoral bifurcation.

### Data analysis

Analyses of both the carotid artery and abdominal aorta IMT were performed using “Coast,” an in‐house code programmed in Matlab (R2022b, The Mathworks Inc., Natick, MA, USA) that uses a template registration segmentation algorithm and has been validated against manual measurements. For carotid analysis, three to five end‐diastolic images were used to calculate the mean and maximum IMT from a 4–10 mm segment of the far wall, measured 1 cm proximal to the carotid bulb. For aortic analysis, the same number of end‐diastolic images was used to calculate the mean and maximum IMT from a 10‐mm segment of the posterior wall. One or both of two graders, blinded to subject status, measured all IMTs. We measured the minimum carotid artery vessel diameter, distensibility [(maximum diameter − minimum diameter)/minimum diameter × 100%], and compliance [(maximum diameter − minimum diameter)/brachial pulse pressure]. These parameters could not be obtained from images of the abdominal aorta due to image quality. Intra‐class correlation (ICC) for inter‐rater reliability was 0.74 and 0.95 for cIMT and aIMT, respectively. The ICC for intra‐rater reliability was 0.84 and 0.98 for cIMT and aIMT, respectively.

### Statistical analysis

Analysis was performed using R software (version 4.2.2, R Foundation, Vienna, Austria). For continuous data, mean (SD) or median (interquartile range with *Q*1, *Q*3) were reported. Categorical data were summarized by count and percentage of nonmissing data. Unadjusted comparisons were based on the two‐sample *t*‐test or Wilcoxon rank sum test. Cardiovascular measures were compared using linear regression models, and quantile regression models (with bootstrapped *P*‐values) for non‐normally distributed outcomes. Models were adjusted for age, sex, BMI *z*‐score, BP (apart from PWV), and LDL cholesterol.

## Results

We enrolled 81 participants with IBD and 82 control participants. Of the IBD participants, 40 had CD (22 [55%] male, 14.58 [2.98] years), 40 had UC (17 [42%] male, 14.32 [2.84] years), and 1 had IBDU. Compared with controls, children with IBD were older, had a higher BMI, higher waist‐to‐hip ratio, lower total cholesterol, lower HDL cholesterol, higher CRP, and a higher proportion of children were post‐pubertal (Table 1). Participants with CD had a median (IQR) disease duration of 3.01 (1.56–4.35) years and those with UC had a median (IQR) of 2.08 (1.02–4.53) years. Eighteen (45%) of the patients with CD and 2 (5%) with UC were on biologic therapy, while 20 (50%) with CD and 19 (45%) with UC were on an immunomodulator (see Supplementary Data).

**Table 1:** Characteristics of control and inflammatory bowel disease participants

Variable	Control (*n* = 82)	IBD (*n* = 81)	*P*‐value
Age at interview (years)	11.88 (3.19)	14.45 (2.88)	<0.001
Time since diagnosis (years)	‐	2.49 [1.23, 4.38]	‐
Sex—Male	40 (49%)	40 (49%)	>0.99
BMI (kgm^−2^)	19.10 (3.22)	20.71 (3.96)	0.005
BMI *z*‐score	0.39 (0.97)	0.23 (1.12)	0.335
Waist hip ratio	0.81 (0.06)	0.83 (0.06)	0.021
Mean arterial blood pressure (mmHg)	80.54 (7.59)	82.10 (8.11)	0.208
Systolic blood pressure (mmHg)	113.8 (9.7)	116.6 (10.7)	0.082
Diastolic blood pressure (mmHg)	65.4 (7.0)	67.0 (6.9)	0.146
Triglycerides (mmol/L)	0.78 (0.32)	0.86 (0.36)	0.133
Total cholesterol (mmol/L)	4.23 (0.70)	3.96 (0.71)	0.017
LDL cholesterol (mmol/L)	2.33 (0.62)	2.21 (0.61)	0.199
HDL cholesterol (mmol/L)	1.54 (0.40)	1.36 (0.31)	0.002
Glucose (mmol/L)	4.36 (0.43)	4.42 (0.39)	0.316
High‐sensitivity CRP (mg/L)	0.3 [0.15–0.6]	0.8 [0.3–2.5]	<0.001
Post pubertal (*n* [%])	22 (27%)	51 (63%)	<0.001

For continuous data, mean (SD) or median [*Q*1, *Q*3] is reported. Categorical data are summarized by count and percentage of non‐missing data.

BMI, body mass index; CRP, C‐reactive protein; HDL, high‐density lipoprotein; IBD, inflammatory bowel disease; LDL, low‐density lipoprotein.

Unlike previous studies,^11^, ^12^ we analyzed arterial structure and function of children with UC and CD separately, which are provided in the Supplementary Data. Once we established there was no difference between the two groups, we were able to pool the results to increase statistical power. After adjusting for age, sex, BMI *z*‐score, BP, and LDL cholesterol, there was no difference in measures of arterial structure and function in children with IBD compared with controls (Table 2).

**Table 2:** Cardiovascular measures in participants with inflammatory bowel disease *vs* controls—unadjusted and adjusted†

Variable	Control *n* = 82	IBD *n* = 81	Unadjusted		Adjusted†	
			Difference (95% CI)	*P*‐value	Difference (95% CI)	*P*‐value
Mean cIMT (mm)‡	0.47 (0.05)	0.47 (0.05)	0 (−0.01, 0.02)	0.788	0 (−0.02, 0.01)	0.56
Maximum cIMT (mm) ‡	0.54 (0.05)	0.54 (0.05)	0 (−0.01, 0.02)	0.654	0 (−0.02, 0.01)	0.56
Delta diameter (mm) ‡	0.95 (0.16)	0.95 (0.19)	−0.01 (−0.07, 0.05)	0.815	0 (−0.06, 0.07)	0.89
Mean diameter (mm) ‡	6.28 (0.46)	6.62 (0.38)	0.35 (0.21, 0.49)	<0.001	0.01 (−0.06, 0.09)	0.77
Mean diastolic cIMT Far (mm) ‡	0.49 (0.05)	0.49 (0.05)	0 (−0.02, 0.02)	0.994	0 (−0.02, 0.01)	0.78
Max diastolic cIMT Far (mm) ‡	0.57 (0.05)	0.57 (0.05)	0 (−0.02, 0.02)	0.962	0 (−0.02, 0.02)	0.85
Mean aIMT (mm) §	0.54 (0.08)	0.58 (0.09)	0.03 (0, 0.06)	0.027	0.02 (−0.02, 0.05)	0.363
Maximum aIMT (mm) §	0.63 (0.09)	0.67 (0.10)	0.03 (0, 0.07)	0.044	0.02 (−0.02, 0.05)	0.379
Pulse wave velocity (m/s) ¶	4.43 (0.65)	4.71 (0.75)	0.28 (0.06, 0.5)	0.012	−0.04 (−0.24, 0.16)	0.671
Carotid artery distensibility (%)¶	16.01 [14.02–18.46]	15.81 [13.43–17.97]	−0.12 (−1.45, 0.83)	0.858	0.45 (−0.46, 1.31)	0.494
Carotid artery compliance ×10^−2^ (mm/mmHg) ¶	2.07 [1.82–2.31]	1.94 [1.75–2.26]	−0.13 (−0.25, −0.06)	0.056	0.08 (−0.07, 0.22)	0.401

^†^ Adjusted for age, sex, BMI *z*‐score, blood pressure (mean arterial pressure), and LDL cholesterol.

^‡^ Also adjusted for minimum carotid diameter.

^§^ Also adjusted for average aortic diameter.

^¶^ Not adjusted for blood pressure (mean arterial pressure).

For continuous data, if normally distributed then the mean (SD) is reported, otherwise if non‐normally distributed (i.e. skewed), then the median [*Q*1, *Q*3] is reported.

cIMT = carotid intima‐media thickness, aIMT = aortic intima‐media thickness.

Children with CD had a lower waist‐to‐hip ratio than children with UC, but otherwise had comparable participant characteristics (see Table S1, Supporting information). Following adjustment, there was no difference in parameters of vascular structure or function between the two IBD subtypes (see Table S2).

## Discussion

In this single‐center, cross‐sectional case–control study, we found no evidence of differences in measures of cardiovascular structure or function in children with IBD compared with healthy controls. In addition, we did not observe any differences between those with CD and UC. Adverse cardiovascular measures in those with IBD may require a longer disease duration to become evident and may reflect whether a child with IBD has well‐controlled disease or ongoing inflammatory activity. For example, elevated cfPWV in adults with IBD was shown to reverse over time with adequate disease control.^19^

There are few prior studies that have investigated cardiovascular measures in children with IBD.^11^, ^12^, ^14^ One study found that children with IBD had increased cIMT compared with controls.^11^ Other parameters of vascular structure and function were not assessed.^11^ In another study with a younger cohort with a shorter disease duration, mean aIMT was higher in IBD patients than controls, with no difference in cIMT.^12^ Neither study adjusted for potential confounders. cfPWV has not previously been shown to differ in children with IBD compared with controls,^14^, ^15^ consistent with our findings.

Our study's limitations include its cross‐sectional nature, and lack of standardized data on disease classification, severity, and disease activity including fecal calprotectin and erythrocyte sedimentation rate. Disease activity indices are limited by only reflecting a single time point. Our sample size was insufficient to allow sensitivity analyses by treatment modality. Despite these limitations, to the best of our knowledge, this is one of the largest cohorts of children with IBD to be investigated for arterial structure and function. Our study is the first in children to measure and adjust for major confounding, which may explain the conflicting results in the literature to date.

We did not show any differences in arterial structure and function in children with IBD compared with healthy controls. While pre‐atherosclerotic changes have been reported in patients with IBD in early adulthood, it likely takes years of poorly controlled disease with chronic inflammation for these changes to develop. IBD diagnosed in childhood may present a window of opportunity to actively reduce cardiovascular risk and promptly induce sustained remission, to potentially prevent future adverse cardiovascular outcomes. Longitudinal studies, over a prolonged duration and with a larger sample size, are required to confirm our findings and inform preventative strategies and intervention targets.

## Ethical approval

This study was approved by the RCH Human Research Ethics Committee (reference number 33065).

## Patient consent

Informed consent for participation in the study was obtained from participants' parent or guardian.

## Supporting information


**Data S1** Supporting information.

## Data Availability

The data that support the findings of this study are not openly available, and de‐identified data can be made available from the corresponding author upon reasonable request.
