# Supplementary material for: Arterial structure and function in children with inflammatory bowel disease
Source: JGH Open. 2024 Jun 3;8(6):e13100. doi: 10.1002/jgh3.13100 (PMC11145743; doi:10.1002/jgh3.13100)
Supplement: Supplementary file 1 — Data S1 Supporting information. [file JGH3-8-e13100-s001.docx]

**Supplementary data**

18 (45%) of those with CD were on biologic therapy (17 on infliximab, and 1 on adalimumab), and 16 (89%) of those participants were on combination therapy with an immunomodulator (10 on azathioprine, and 6 on methotrexate). 20 (50%) participants with CD were on an immunomodulator without biologic therapy (15 on azathioprine, 5 on methotrexate), while the 2 remaining participants were on 5-aminosalicylic acid (5-ASA) therapy only. Of the participants with UC, 2 (5%) were on infliximab therapy in combination with azathioprine, 19 (45%) on azathioprine, and 19 (45%) on 5-ASA only. Data are presented as mean (SD), or median [interquartile range Q1, Q3].

**Table S1: Patient characteristics by IBD sub-classification**

|  | | | | **P-value - Comparison to Control (Table1)** | |
| --- | --- | --- | --- | --- | --- |
| **Variable** | **Crohn's disease**  **N= 40** | **Ulcerative colitis**  **N=40** | **P-value** | **Crohn's disease** | **Ulcerative**  **colitis** |
| Age at interview (years) | 14.58 (2.98) | 14.34 (2.84) | 0.711 | <0.001 | <0.001 |
| Time since diagnosis (years) | 3.01 [1.56, 4.35] | 2.08 [1.02, 4.53] | 0.173 | - | - |
| Sex - Male | 22 (55%) | 17 (42%) | 0.371 | 0.566 | 0.565 |
| BMI (kgm^-2^) | 20.48 (4.15) | 21.03 (3.81) | 0.537 | 0.069 | 0.007 |
| BMI z-score | 0.09 (1.26) | 0.41 (0.95) | 0.205 | 0.181 | 0.948 |
| Waist hip ratio | 0.81 (0.06) | 0.84 (0.06) | 0.049 | 0.454 | 0.004 |
| Mean arterial blood pressure (mm Hg) | 82.47 (7.61) | 81.75 (8.76) | 0.695 | 0.192 | 0.458 |
| Systolic blood pressure (mmHg) | 118.2 (10.7) | 115.15 (10.8) | 0.215 | 0.033 | 0.503 |
| Diastolic blood pressure (mmHg) | 67.5 (5.8) | 66.47 (7.9) | 0.527 | 0.087 | 0.462 |
| Triglycerides (mmol/L) | 0.86 (0.35) | 0.86 (0.37) | 0.912 | 0.186 | 0.251 |
| Total cholesterol (mmol/L) | 4.06 (0.83) | 3.89 (0.55) | 0.298 | 0.272 | 0.005 |
| LDL cholesterol (mmol/L) | 2.27 (0.70) | 2.17 (0.48) | 0.434 | 0.662 | 0.112 |
| HDL cholesterol (mmol/L) | 1.39 (0.35) | 1.33 (0.28) | 0.374 | 0.040 | 0.001 |
| Glucose (mmol/L) | 4.48 (0.39) | 4.38 (0.39) | 0.275 | 0.138 | 0.774 |
| High-sensitivity CRP (mg/L) | 1.2 [0.45, 4.15] | 0.55 [0.27, 1.92] | 0.071 | <0.001 | 0.009 |
| Post pubertal | 26 (65%) | 25 (62%) | >0.99 | <0.001 | <0.001 |

**Table S2: Cardiovascular measures in participants with Crohn’s disease vs. ulcerative colitis**

|  | | | **Unadjusted** | | **Adjusted†** | |
| --- | --- | --- | --- | --- | --- | --- |
| **Variable** | **Crohn’s disease**  **N=40** | **Ulcerative colitis**  **N=40** | **Difference (95% CI)** | **P-value** | **Difference (95% CI)** | **P-value** |
| Mean cIMT (mm)* | 0.47 (0.06) | 0.46 (0.03) | -0.01 (-0.03, 0.01) | 0.479 | 0 (-0.02, 0.02) | 0.975 |
| Maximum cIMT (mm)* | 0.54 (0.06) | 0.54 (0.03) | -0.01 (-0.03, 0.02) | 0.601 | 0 (-0.02, 0.03) | 0.737 |
| Delta Diameter (mm)* | 0.90 (0.17) | 0.99 (0.21) | 0.09 (0, 0.18) | 0.057 | 0.08 (-0.01, 0.17) | 0.079 |
| Mean Diameter (mm)* | 6.53 (0.33) | 6.72 (0.42) | 0.18 (0, 0.36) | 0.051 | -0.02 (-0.11, 0.07) | 0.689 |
| Mean Diastolic cIMT Far (mm)* | 0.49 (0.06) | 0.48 (0.03) | -0.01 (-0.03, 0.02) | 0.565 | 0 (-0.02, 0.02) | 0.922 |
| Max Diastolic cIMT Far (mm)* | 0.57 (0.07) | 0.56 (0.03) | -0.01 (-0.03, 0.02) | 0.675 | 0 (-0.02, 0.03) | 0.760 |
| Mean aIMT (mm)** | 0.57 (0.10) | 0.58 (0.08) | 0.01 (-0.03, 0.06) | 0.586 | 0.01 (-0.05, 0.06) | 0.809 |
| Maximum aIMT (mm)** | 0.66 (0.11) | 0.68 (0.09) | 0.02 (-0.04, 0.07) | 0.529 | 0.01 (-0.06, 0.07) | 0.801 |
| Pulse wave velocity (m/s) ‡ | 4.69 (0.65) | 4.73 (0.86) | 0.03 (-0.3, 0.37) | 0.844 | -0.01 (-0.32, 0.29) | 0.937 |
| Carotid artery distensibility (%)‡ | 15.39 [12.85 - 17.29] | 15.92 [13.95 - 18.22] | 0.53 (-0.74, 2.72) | 0.602 | 0.911 (-0.91, 1.81) | 0.397 |
| Carotid artery compliance x 10^-2^ (mm/mm Hg)‡ | 1.88 [1.71 - 2.01] | 1.98 [1.82 - 2.33] | 0.07 (-0.07, 0.32) | 0.547 | 0.085 (-0.02, 0.29) | 0.492 |

*cIMT = carotid intima media thickness, aIMT = aortic intima media thickness*

*Notes:* *For continuous data, if normally distributed then the mean (SD)is reported, otherwise if non-normally distributed (i.e., skewed) then the median [Q1, Q3] is reported.*

*†Adjusted for age, sex, BMI z-score, blood pressure (mean arterial pressure) and LDL cholesterol*

**Also adjusted for minimum carotid diameter*

***Also adjusted for average aortic diameter*

*‡ Not adjusted for blood pressure (mean arterial pressure)*
